# Design and Synthesis of Porous Organic Polymers: Promising Catalysts for Lignocellulose Conversion to 5-Hydroxymethylfurfural and Derivates

**DOI:** 10.3390/polym15122630

**Published:** 2023-06-09

**Authors:** Lei Yang, Lishu Shao, Zhiping Wu, Peng Zhan, Lin Zhang

**Affiliations:** 1 Ministry of Forestry Bioethanol Research Center, School of Materials Science and Engineering, Central South University of Forestry and Technology, Changsha 410004, China; 2 Hunan International Joint Laboratory of Woody Biomass Conversion, Central South University of Forestry and Technology, Changsha 410004, China

**Keywords:** porous organic polymer, biomass conversion, lignocellulose, 5-hydroxymethylfurfural, eco-friendly catalysts

## Abstract

In the face of the current energy and environmental problems, the full use of biomass resources instead of fossil energy to produce a series of high-value chemicals has great application prospects. 5-hydroxymethylfurfural (HMF), which can be synthesized from lignocellulose as a raw material, is an important biological platform molecule. Its preparation and the catalytic oxidation of subsequent products have important research significance and practical value. In the actual production process, porous organic polymer (POP) catalysts are highly suitable for biomass catalytic conversion due to their high efficiency, low cost, good designability, and environmentally friendly features. Here, we briefly describe the application of various types of POPs (including COFs, PAFs, HCPs, and CMPs) in the preparation and catalytic conversion of HMF from lignocellulosic biomass and analyze the influence of the structural properties of catalysts on the catalytic performance. Finally, we summarize some challenges that POPs catalysts face in biomass catalytic conversion and prospect the important research directions in the future. This review provides valuable references for the efficient conversion of biomass resources into high-value chemicals in practical applications.

## 1. Introduction

Facing the current global energy and environmental problems, it is urgent to replace fossil energy with renewable and environmental alternatives to produce a series of high-value chemicals and clean fuels. The effect of biomass resources on these two aspects can effectively overcome the dependence on fossil energy [1,2]. Lignocellulose, the main structural component of plants, is by far the most abundant renewable resource. It is capable of being converted in significant quantities into biofuels, such as bioethanol [3], and other commercial chemicals, such as 5-hydroxymethylfurfural (HMF), sugars, and phenols.

**Figure 1:**
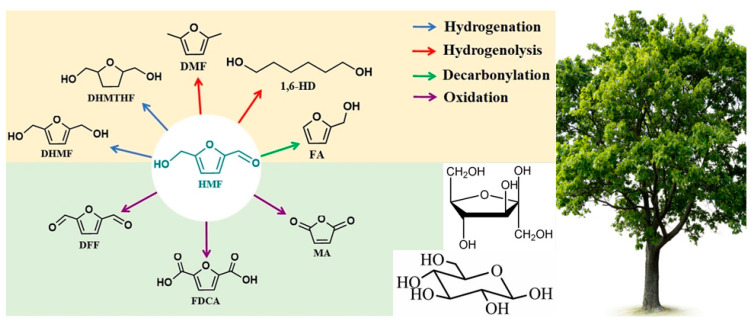
Partial transformation route of HMF [7].

HMF is a biological platform molecule that can be produced from lignocellulose, that is, lignocellulose is hydrolyzed into six-carbon sugars (glucose and fructose) and further dehydrated [4]. It is an intermediate as well as a raw material for many important chemicals and can be converted into high value-added chemicals by oxidation, hydrogenation, polymerization, and ring-opening reactions by its own furan rings, aldehyde groups, and hydroxyl groups. Some of the products are shown in Figure 1 [5,6,7], such as cross-linking agents of polyvinyl alcohol (PVA) for battery manufacturing [8] and γ-valerolactone (GVL), which produces high value-added fuels. In particular, 2, 5-furanodicarboxylic acid (FDCA), for the substitution of petroleum-based aromatic compounds, can be produced by the oxidation path of HMF [9]. It can be polymerized with ethylene glycol to produce polyrthylene furandicarboxylate (PEF), which can replace polyethylene terephthalate (PET) synthesized from petroleum-based compounds as precursors [10]. Furthermore, other polyester products synthesized by FDCA are also promising, such as bio-based copolyesters, which can be used as 3D printing materials [11].

The existing catalytic preparation of HMF can be roughly divided into two categories. Homogeneous catalysts are represented by inorganic acid [12], ionic liquid [13], metal salt [14], etc. In this kind of catalyst system, although the active center of the catalyst is uniform and the structure is clear, the separation of the catalyst in the reaction mixture is difficult and may be accompanied by side reactions, and the recovery performance of the catalyst is greatly reduced. Consequently, from an industrial perspective of product separation and equipment maintenance, etc., a heterogeneous catalyst is more advantageous. Due to various defects and the local environment of active sites, the coexisting environment of multiple active sites created is beneficial to improve the catalytic efficiency, but attention should also be paid to product selectivity. Some researchers have used metal oxides [15], metal carbide [16], functional carbon materials [17], etc., as catalysts for achieving HMF. The study of the catalytic active site, pore size, solvent environment, and other aspects is also often carried out to improve the catalytic efficiency of the whole reaction. Similarly, the above discussion of catalysts is also applicable to the catalytic conversion of HMF.

Supported catalysts, as typical heterogeneous catalysts, have naturally become a hot option for catalyst structures, and can not only be combined with precious metals to further improve their efficiency but can also achieve efficient conversion by combining non-precious metals [18] or even environmentally friendly non-metallic components [19] with suitable carriers. Here, the selection of the catalyst support and active ingredients (sites) are very important. For the support, it is often a class of a solid porous material with a high surface area, suitable pore size and distribution, and adequate pore volume. Of course, some porous supports can also be directly used as the catalyst or cocatalyst themselves [20].

A porous material is a kind of functional material composed of interconnected or closed holes. According to the pore size, they can be divided into micropore materials (<2 nm), mesoporous materials (2–50 nm), and macropore materials (>50 nm). We are familiar with porous materials such as metal-organic frameworks (MOFs)**,** porous organic polymers (POPs), activated carbon, silica, clay, and so on [21]. They have been used in a wide range of fields, nowadays, such as porous silicon for the manufacture of photoelectric components, porous membranes, and porous adsorbents for separation, thermal, and sound insulation materials, biomedical materials, and so on [22]. Porous materials can not only improve the utilization of the volume of material based on the stability and screening characteristics of the pores but also provide more space for the contact between active sites and reaction substrates, which greatly promotes the performance of catalysts [23]. When metal components are the important active sites, a supported porous catalyst can ensure high efficiency, but there are also side reactions, low selectivity, and metal leaching into the product [24]. Hence, as one of the hot topics in catalysis research, nonmetallic porous carbon materials have the advantages of low density, no metal leakage pollution, good structural ductility, and strong acid and alkali resistance [25]. They are often used as a kind of catalytic material with great potential for energy storage and conversion [26] and can realize efficient conversion through the functionalization and loading of active components. They have excellent application in catalytic biomass valorization and environmental remediation [27]. However, since porous carbon materials are usually prepared by high-temperature pyrolysis, it is relatively difficult to predict and control their structural features [28]. In addition, their energy consumption and equipment cost are relatively high. Therefore, the development of high-performance catalysts with well-defined synthetic structures, low cost, and environmentally friendly features is of paramount importance, especially for the catalytic field of the preparation and conversion of HMF.

In the pursuit of the desired characteristics mentioned above, researchers are starting to pay more attention to the gradual update of catalyst structures from an inorganic skeleton to an organic skeleton [29]. At present, POPs are widely used in the field of catalysis due to their excellent physical and chemical properties, good thermal stability, large specific surface area, controllable pore size, and designable chemical structure [30]. Specifically, in the fields of photocatalysis [31,32] and photoelectrocatalysis [33], they have shown good performance. Compared to other inorganic porous materials, the advantages of POPs is that, first of all, they are mainly composed of light elements—C, H, O, N, and B—and have low skeletal density [34]. Secondly, they have rich organic synthesis strategies and various types of monomers, and they are connected by high-energy covalent bonds, with inherent porosity and high physical and chemical stability. Therefore, they can effectively avoid the above-mentioned catalyst recovery and product separation problems, and in the design of the catalyst structure, compared to the popular carbon materials, more accurate control can be achieved [35]. They can even achieve high-efficiency metal-free catalytic conversion by virtue of their special structure. Depending on the framework features and synthesis method, POPs mainly include covalent organic frameworks (COFs) [36], porous aromatic frameworks (PAFs) [37], hyper-cross-linked polymers (HCPs) [38], conjugated microporous polymers (CMPs) [39], and other types. They not only have many applications in catalysis but also certain advantages and effects on gas storage, molecular separation, drug delivery, and sensing [40,41]. Likewise, the heterogeneous catalytic system for the preparation of HMF from biomass resources, and the subsequent conversion of HMF discussed in this paper have also shown good performance and have provided important knowledge for subsequent research. It is essential and significant to explore the potential application of POPs in the catalytic conversion of biomass. This paper mainly considers the preparation of biomass-based HMF, focusing on the catalytic conversion of fructose and the subsequent transformation of different types of POPs catalysts. In addition, we explore the unique advantages of the specific structure of the catalyst on the reaction, analyze the problems and research direction of POPs in the catalytic process of biomass, and predict more uses of POPs in the field of catalysts.

## 2. Synthesis and Structure of POPs

**Figure 2:**
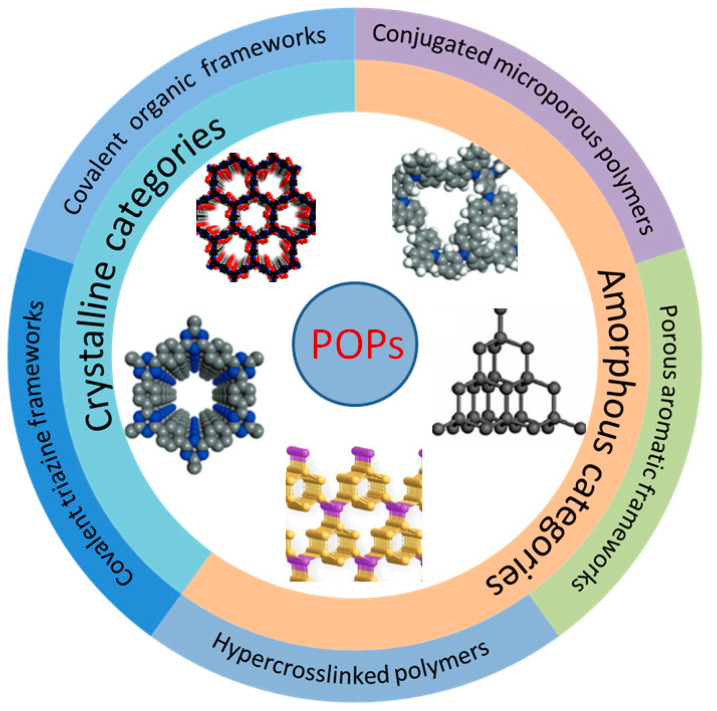
Partial classification of POPs.

A POP is a kind of organic porous functional material with a microporous and mesoporous structure. As shown in Figure 2, according to their structural characteristics, they can be divided into amorphous categories (representing PAFs, CMPs, and HCPs) and crystalline categories (representing COFs and covalent triazine frameworks (CTFs) [42]). POPs can be classified into different categories and achieve various functions based on different monomers and synthesis routes, such as Suzuki coupling [43], the Friedel–Crafts alkylation reaction [44], Schiff-base condensation [45], and Hagihara cross-coupling [46], etc. Their unique physical and chemical properties have enabled POPs to achieve good results in adsorption, catalysis, sensing, battery production, energy storage, biomedicine, film material, and other fields [47,48,49,50,51].

COFs are emerging crystalline porous molecular materials composed of organic molecules linked by strong covalent bonds (such as B-O, C=N, C-N, C=C, etc.) and are composed of two main components: the covalent bond skeleton and the modifiable functional group. COFs were first synthesized and reported by Omar Yaghi et al. in 2005 [52]. On account of the large specific surface area, periodicity, and regular and adjustable channel structure, the functional surface and the inner wall of the channel can be used for catalysis after loading the active component. From the perspective of topological structure, COFs can be divided into two categories. Two-dimensional COFs have a unique, layered morphology, namely conjugate planes and π-π structures stacked between layers, which form regular channels in the longitudinal direction. Three-dimensional COFs are connected by covalent bonds to form a network, so they are highlighted by many open sites and low density [53]. Both structures can ensure the stability of COF crystallinity by placing active sites into their own structures, which is also very representative of heterogeneous catalysis. The polymerization of COFs is generally carried out according to the principles of dynamic covalent chemistry and reticular chemistry, and the final product is determined by the thermodynamic stability of the substrate. However, due to their dependence on dynamic reversible reaction synthesis, their stability is lower than that of other POP subclasses, and due to the limitations of reversible reaction types, their synthesis scheme and structural diversification need to be developed.

Unlike COFs, PAFs are usually formed through irreversible coupling reactions, such as Ullmann coupling. The term PAFs was first proposed in 2009 [54]. A diamond-like structure was formed by a rigid benzene ring, as shown in Figure 2. Interestingly, in addition to a large internal surface area, a very strong skeleton formed by a bottom-up C-C bond between aromatic structural units enabled excellent structural stability even under some extreme conditions [55]. In summary, there are three important aspects that determine the synthetic diversity and performance of PAF materials, which are symmetrical structure-containing monomers, efficient coupling reactions, and the topological design of the framework. By modifying the functional group of the framework unit, the specific function of a certain substance can be realized. For example, the capture of CO_2_ can be realized through the amino group on the aromatic structure [56]. At the same time, its adjustable physical and chemical properties and large specific surface area can also be well used in biomass catalysis.

**Figure 3:**
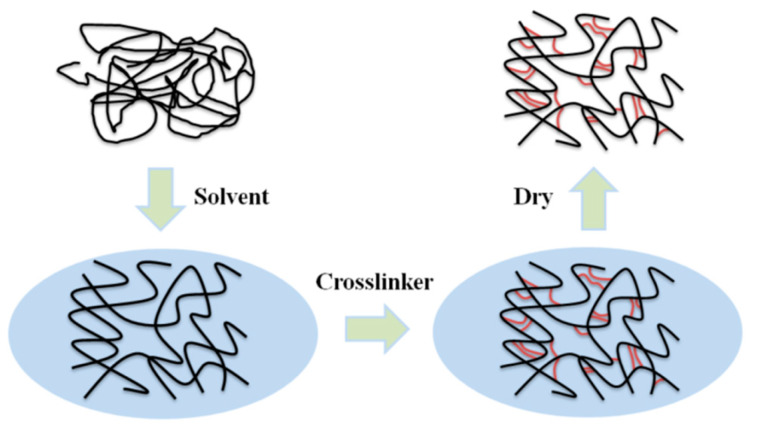
Schematic diagram of hyper-cross-linked process.

Porous materials with good structural properties often have a higher preparation cost, but HCPs differ from other types of POPs in that they can be directly synthesized by post-modification easily through Friedel–Crafts (FC) reactions in mild conditions [57]. As a kind of polymer material with a permanent microporous structure, HCPs can achieve a specific structure depending on the choice of monomer, cross-linking agent, and appropriate reaction routes. Figure 3 shows the specific hyper-cross-linked process. At present, there are three main synthesis methods: post-cross-linking [58], direct polycondensation [59], and external cross-linking [60]. Their large surface area, light weight, and mild prepared condition make HCPs particularly outstanding in the fields of adsorption and catalysis.

**Figure 4:**
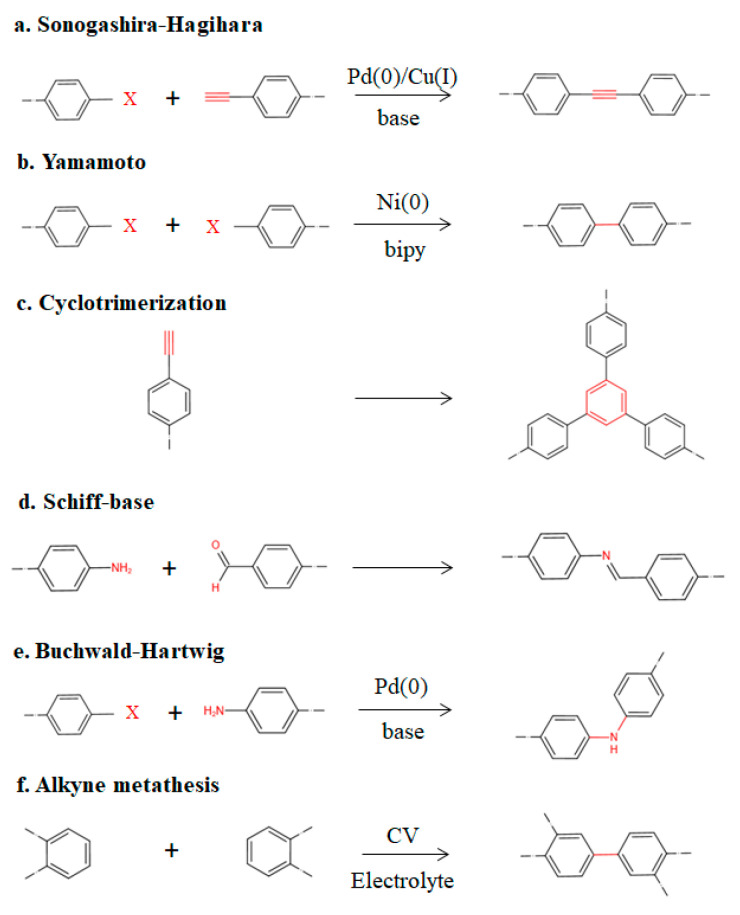
Partial synthetic strategies of CMPs (X = Cl, Br, I, OTf). (**a**) Sonogashira–Hagihara, (**b**) Yamamoto, (**c**) cyclotrimerization, (**d**) Schiff-base, (**e**) Buchwald–Hartwig, (**f**) alkyne metathesis.

Analogously, conjugated microporous polymers (CMPs) are porous organic materials that combine an extended π conjugated structure with a permanent microporous framework, and they are basically three-dimensional conjugated networks [61]. CMPs can be formed out of several different monomers through coupling reactions, and they can be tuned by changing the geometry of the monomer or combining it with other atoms. The substitution of aromatic structures by alkenes, alkynes, halogens, aldehydes, or other groups is already available for the synthesis of CMPs, and many more groups are waiting to be discovered. Figure 4 shows some of the various routes. As a result, CMPs have become the broadest subcategory of POP material development. The CMP catalysts that have directly introduced active groups into the framework are convenient for recycling due to their insoluble properties, and great progress has been made in the field of heterogeneous catalysis in recent years [62]. However, highly cross-linked networks and π-conjugated rigid structures have also greatly reduced the machinability of CMPs and limited their application scope.

To sum up, POPs have become an important part of porous supports and catalysts with their excellent physical and chemical properties, and each subclass has also had good results in the catalytic conversion of biomass after the functionalization and loading of active components due to their unique structural properties.

## 3. Catalytic Conversion of Fructose on POPs

Natural wood after a simple treatment can become non-toxic, tasteless, and pollution-free lignocellulose, and the use of biomass resources is mostly concentrated on the treatment [63]. The hydrolysis of cellulose into glucose is the starting point of its high-value transformation. Glucose can not only produce the most widely used industrial raw material ethanol, but also isomerize to fructose for producing lactic acid, HMF, and other important platform compounds. Hemicellulose can also be converted into furfural, xylitol, and other compounds after acid hydrolysis. Lignin is the most abundant renewable aromatic compound in nature and can also be used in the production of fuel and high-value chemicals. How to overcome the complexity and obstinacy of its structure and realize its efficient utilization is necessary to continue to study. Using efficient catalytic technology to transform lignocellulose into high value-added chemical platform compounds and derive downstream green chemicals on the basis of platform molecules will accelerate the arrival of the era of carbon neutrality. This section focuses on the conversion path of lignocellulose to fructose and, subsequently, HMF.

**Figure 5:**
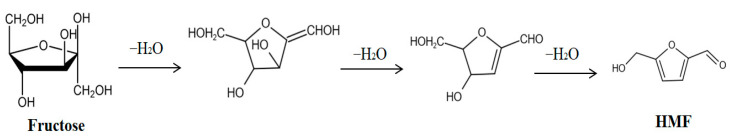
Conversion mechanism of fructose in preparation of HMF [74].

At present, researchers have used cellulose as a raw material to prepare HMF by a one-pot method [64] or a multi-step method, and others have also used mannose with the same intermediate as glucose as raw materials and other polysaccharides for conversion [65]. Here, we mainly introduce two preparation paths for HMF. One is directly preparing it by removing three molecules of water from glucose. The second is by using fructose as a starting point under certain conditions to generate a specific intermediate and gradually removing three molecules of water collocation [66]. Wang et al. proposed the mechanism of C_6_ dehydration to prepare HMF, and reasonably concluded that the precondition for glucose to HMF was to convert it into fructose first [67]. Structurally, glucose with a pyran ring structure was more stable and more difficult to convert into HMF than fructose with a furan ring structure [68]. In a specific study on the preparation of HMF from lignocellulose-derived fructose, the results and analysis also verified the reliability of the above transformation mechanism [69]. If the efficient catalytic technology can be used to convert biomass resources into high value-added chemical platform compounds, and these platform molecules can derive downstream green chemicals, this will accelerate the arrival of the era of carbon neutrality [70]. Moreover, fructose, as an isomer of glucose, is one of the most common hexoses. It is an excellent biomass product and can be converted into polyester monomers and additives [71] through a series of transformations. It has also been widely used in food and medicine production [72]. Therefore, most studies have designed green and efficient catalytic systems using fructose as a raw material. Among them, the most popular route has been the catalytic reaction of dehydration to prepare HMF. Figure 5 shows the conversion mechanism of fructose to prepare HMF by removing three molecules of water with an acid catalyst [73].

In a reaction system with POPs as the main component of the catalyst, strong covalent bonds were created between the monomers to ensure the stability of the structure in a solvent, ensuring that the substrate could fully contact the active site in the catalytic reaction [74]. After multifunctional acid functionalization, it had corresponding groups, which ensured high conversion and selectivity. The subclasses of POPs have gained favor among scientists. For example, regarding amorphous PAFs and HCPs, the former has an extremely high surface area, while the latter has economical and mild synthesis conditions, both of which have achieved extremely high conversion rates in biomass conversion, especially fructose. Periodic COFs, with their unique crystal structure and synthesis methods, have not only been applied in traditional thermal catalysis but also have the possibility of realizing efficient biomass conversion through electrocatalysis, and hence, its development was worth our deep thinking.

Due to the fact that most COFs are synthesized by reversible reactions, they are not only less stable than other POP types but also limited to the symmetry of the structural monomers. Therefore, they need to be developed to improve their synthesis scheme and topological structure to improve their stability [75]. Peng et al. prepared irreversible enol-ketone intervariant structures with significant chemical stability by an alkali condensation reaction and treated with 1, 3, 5-triformyl-phloroglucinol (TFP) and 2, 5-diaminobenzene sulfonic acid (DABA) to obtain a solid acid catalyst, called TFP-DABA [76]. Two-dimensional COFs enhanced the degree of sulfonation and increased the number of Brønsted acid sites by virtue of their pore structure formed by the accumulation of layered structures. With this characteristic, the catalytic system was reacted in dimethyl sulfoxide (DMSO) at 100 °C for 12 h to obtain 97% HMF from fructose and 65% of its subsequent oxidation product 2, 5-formylfuran (DFF). No significant decrease in activity was observed after three cycles. Most fructose catalytic conversion systems with POPs as a catalyst could not get rid of their dependence on DMSO, which stabilizes HMF and inhibits the occurrence of side reactions but is also a very harmful solvent to the environment.

**Figure 6:**
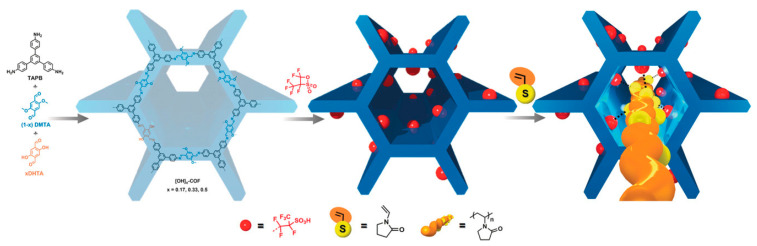
The concept of changing the local environment of the active site in porous materials by inserting linear polymers and the synthesis strategy of PVP@[SO_3_H]_0.17_-COF [77].

**Figure 7:**
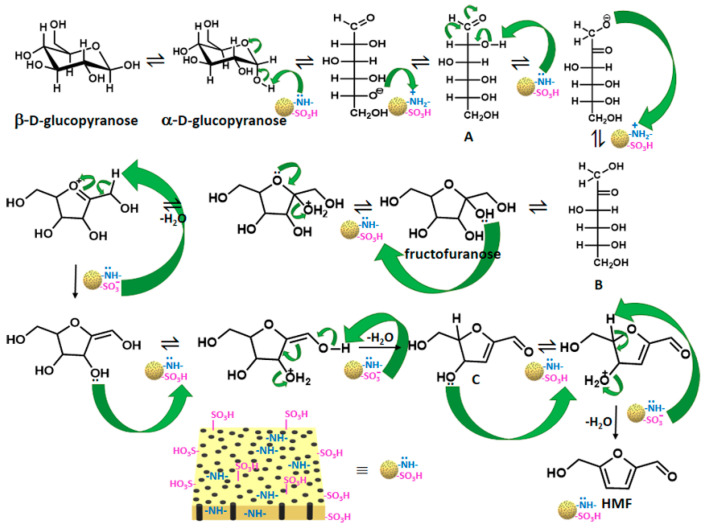
Mechanistic pathway for the conversion of glucose and fructose into HMF over bifunctional porous organic polymers [78].

By exploring the influence of non-covalent interactions on catalytic performance, Sun et al. modified the environment around the active site of the catalyst by using the movable and aggregable properties of the groups on a highly flexible linear polymer, and even achieved better conversion efficiency without DMSO. As shown in Figure 6, they prepared a novel COF with large mesoporous channels by the condensation reaction of 1,3,5-tris(4-aminophenyl)-benzene (TPB) and 2,5-dimethoxyterephthalaldehyde (DMTA) [77]. We can clearly see that the perfluoroalkyl chain with sulfonic acid groups was confined to the pore through the reaction of the sultone ring with the hydroxyl group on the COF framework, and then through the hydrogen bond interaction to create the desired solvent environment. When PVP@[SO_3_H]_0.17_-COF was inserted into the highly flexible polymer synthesis catalyst, the fructose conversion rate was greater than 99.5% and the HMF yield was as high as 99.1% at 100 °C for only 30 min. The catalyst could still maintain the fructose conversion rate after five cycles, and the HMF yield only decreased by 2%. The studies carried out by Bhanja et al. were not limited to monocatalytic centers [78]; they used 2,2′-benzidinedisulfonic acid and 4,4′-diaminostilbene-2,2′-disulfonic acid with cyanuric chloride to synthesize catalysts (POPDS and POPSDS) containing sulfonic acid and secondary amine, which became excellent catalysts to prepare HMF. The reaction at 130 °C for 20 min achieved a 98% fructose conversion and 86% and 89% HMF yields, and the catalyst maintained six cycles of experiments. Figure 7 shows the mechanism of the conversion of glucose and fructose into HMF. It can be seen in the figure that the two groups had different functions. The amino group was mainly responsible for the isomerization transformation, while the sulfonic acid group played a role in the subsequent transformation. Their presence guaranteed the high catalytic performance of the reaction.

The synthesis system of COFs endowed them with unique structural properties. In the formation and connection of the covalent bonds, the high synthesis rate sped up the nucleation process in the synthetic system, which led to a great reduction in the crystallinity of the COFs, and made it difficult to obtain single-crystal COFs. This structure made their stability weaker than that of other subclasses. This characteristic was reflected in the catalytic reaction, which shortened the catalyst life and affected the economic and time benefits [79]. Therefore, the development of a new synthetic approach to discuss the stability and crystallization of COFs was worthy of further study.

In addition to increasing the specific surface area and the number of acid sites of the catalyst, the catalytic activity and product selectivity of converting fructose into HMF was also considered due to the low diffusion of fructose molecules in the cellular structure [80]. Subsequently, some PAFs were recently reported for this catalytic reaction. Du et al. used rigid tetrahedrons and triangles to construct porous aromatic framework PAFs to ensure the suitable pore size and the availability of active sites [81]. Their catalyst HO_3_S-POP was highly adapted to the substrate with a high specific surface area and could achieve a 100% fructose conversion within 15 min under microwave heating to 140 °C, and 70% HMF selectivity.

Unlike crystalline framework materials, such as COFs, PAFs are composed of irreversible structures, more attention should be paid to the uniqueness of their topological design. The structure formed by an appropriate coupling reaction can realize high product selectivity. In the local structure, aromatic groups with certain rules can rotate and combine to deform, thus forming structural defects that fit with the reaction system. Therefore, it is necessary to clearly study the framework of PAFs, understand their structural composition in depth, and further broaden the scope of their heterogeneous catalysis.

Although HCPs with a permanent microporous structure cannot be dominant in fructose diffusion, the preparation and application of HCPs with a low threshold is still one of the best choices for porous catalytic materials. Das et al. synthesized the microporous polymer TrzDBTH by the Friedel–Crafts reaction of 2,4,6-tris [4-(bromomethyl)phenyl]-1,3,5-triazine and dibenzothiophene (DBTH), and then sulfonated it with chlorosulfonic acid to obtain a solid acid catalyst, STrzDBTH, with sulfonic acid groups [82]. The complete conversion of fructose and 96.2% yields of HMF were achieved after a 20 min reaction at 140 °C under atmospheric pressure. The catalyst could maintain five reaction cycles. Dong et al. verified the effect of the sulfonation treatment and reaction solvent on the dehydration of fructose into HMF by the self-polymerization of a benzene monomer into HCP-x [83]. An HMF yield of 96.7% was obtained at nearly 140 °C for half an hour, and the catalyst could be cycled more than four times.

Porous organic polymers of other HCP structures with different monomers and synthesis methods have also been used in biomass catalytic conversion, such as porous polytriphenylamine (PPTPA), which was self-polymerized from trianiline and can be used as an adsorbent for aromatic compounds and hydrogen bonding compounds because of its conjugated system and large number of nitrogen atoms [84]. Mondal et al. obtained SPPTPA-1 after sulfonation from the PPTPA-1 material prepared above. Acid–base titration showed that SPPTPA-1 had strong acidity, which could be used as a solid acid catalyst for the preparation of HMF by the dehydration of fructose [85]. The fructose was completely transformed in an organic solvent system at 140 °C for 20 min, and HMF with a yield of 94.6% was obtained. The activity of the catalyst did not decrease significantly after four catalytic cycles. In addition, Sebati et al. formed a solid acid catalyst, SPPTPA, by adding chlorosulfonic acid [86]. In order to further facilitate recycling, they used Fe_3_O_4_ nanoparticles to synthesize the catalyst FeSPPTPA for the fructose preparation of HMF, achieving a higher specific surface area than other catalysts and reaching an HMF yield of 95% at 100 °C for 20 min. In the process of separation and recovery, magnetic characteristics could be effectively used for intelligent processing, and the activity of the catalyst did not decrease obviously in four reaction cycles, which also had a certain reference for subsequent research and innovation.

Sulfonic acid-functionalized materials are widely used in the field of catalysis [87]. In the dehydration reaction of the fructose preparation of HMF, a POP catalyst was almost sulfonated in the synthesis process and contained sulfonic acid sites, which proved able to improve the reaction efficiency. To broaden the research direction, Ravi et al. [88] used phosphate-functionalized POPs for biomass conversion into HMF for the first time. They synthesized the HCP-structured catalyst (B-POP) by replacing the sulfonic acid site with a phosphoric acid site. The reaction was carried out in DMSO at 160 °C for 30 min to obtain a 100% fructose conversion and 85% HMF production. The catalytic effect was further used to convert HMF into the oxidation product DFF and the ring-opening product Levulinic acid (LA). In addition, the fructose conversion rate was only reduced by less than 5% after 10 cycles, and the HMF yield did not change visibly.

Certainly, in the application of POPs as catalysts in the conversion of fructose, there are still some aspects to be investigated, such as avoiding side reactions, reducing the use of raw materials, and making the reaction conditions more moderate, etc. Here, we mainly discuss the influence of the solvent system on the reaction. Table 1 summarizes the catalytic system of fructose conversion into HMF with POPs mentioned in this section as the catalysts. The data show that most of the systems worked under atmospheric pressure, and the working conditions were relatively mild. In about half an hour, they achieved raw material conversion rates of more than 95%, and the product yield was between 70% and 98%. Furthermore, it was not difficult to find that in the conversion of fructose to HMF, the solvent used was mostly DMSO, and in other biomass conversion reactions, it was also commonly used as a solvent [89]. From a green point of view, it is undoubtedly better to reduce the harm of DMSO or not use DMSO in the reaction.

## 4. Catalytic Conversion of HMF on POPs

Climate change and the growing depletion of non-renewable energy are putting enormous pressure on all sectors of the world, forcing economies to scale up to meet demand [90]. The chemical industry, which relies on fossil fuels to run, urgently needs to change this status. As mentioned above, PEF using FDCA as the precursor has the potential to replace PET, but the current process route for FDCA production is not mature, and it has not been able to achieve large-scale production of degradable polyester for practical application [91]. The most possible method for large-scale production is to prepare FDCA by the catalytic reaction of HMF, which can be converted from biomass resources. For different oxidation methods, the catalytic methods for preparing FDCA from HMF can be divided into thermal catalysis [92], photocatalysis [93], electrocatalysis [94], and biocatalysis [95]. According to the current production level, thermal catalysis is the most compatible method in industry. Among the catalysts selected, the most effective ones were those with precious metal components [96]. However, due to the scarcity of precious metals, industrialization can not be realized according to the current industrial level. The catalysis of non-noble metals or even non-metallic components with a porous carrier for the preparation of FDCA from HMF has always been an interest of scholars. Solid acid catalysts with POPs as the carrier have shown considerable potential in this conversion route and other conversion routes of HMF, but there are also some research areas worth improving to enhance the competitiveness of existing catalysts for the conversion of HMF.

Two-dimensional COFs were designed from symmetric and asymmetric topologies to order layered stacked lattice patterns, and regular carrier channels formed along the longitudinal axis, so there is also space for application in the field of electrocatalysis [97]. Since most COFs are non-conductive materials, in order to improve their catalytic efficiency, their unique structures could be used to carbonize the pyrolysis treatment template, and their utilization forms could also be efficiently designed. Cai et al. used nickel acetate, triformyl-phloroglucinol-5,5-diamino-2,2-bipyridine, and toluene sulfonic acid to obtain 2D COF structure-containing films by interfacial crystallization and stamped them onto fluorine-doped tin oxide (FTO) to prepare the catalyst TpBpy-Ni@FTO [98]. This achieved up to a 96% HMF conversion and obtained 58% and 34% FDCA and FFCA oxidation products, respectively. As the first case of electrocatalytic oxidation of HMF with a COF structure, the product yield of this system was not ideal. We considered that the catalyst might be gradually covered by nickel element with low surface electroactivity during the reaction process [99]. Nevertheless, this provides some basis and optimization reference for the subsequent biomass electrocatalysis with a COF structure.

**Figure 8:**
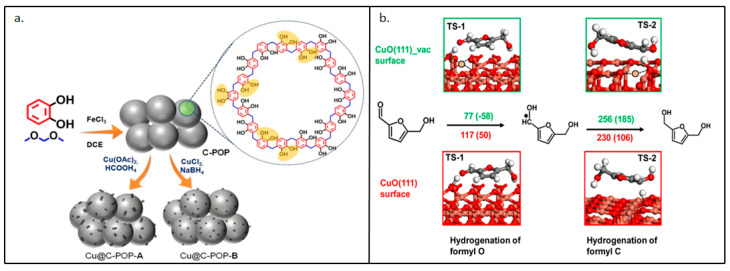
(**a**) Synthetic strategy of as-synthesized catechol-functionalized POP (C-POP), Cu@C-POP-A and Cu@C-POP-B. (**b**) Reaction energy profile of hydrogenation process of HMF conversion to DMHF [107].

FDCA is not the only important substance in the oxidation path of HMF. Others such as DFF, 5-hydroxymethyl-2-furan carboxylic acid (HMFCA), and 5-formyl-2-furan carboxylic acid (FFCA) can also be widely used in the preparation of fine chemicals, pharmaceuticals, chiral catalysis, and polyester industry [100,101,102]. Similarly, the oxidation route is not the only one worth discussing in the utilization of HMF. HMF, known as “the sleeping giant” [103], can activate C=O, C=C, and C-O itself for high-value conversion in green sustainable chemistry. 2,5-dihydroxymethylfuran (DHMF) is an important chemical in the production of artificial fibers and resins and can be produced by the hydrogenation of HMF [104]. Most studies have relied on the synergistic system of Cu^0^ and Cu^+^ to improve the catalytic activity in hydrogenation treatment [105]. However, due to the existence of reducing gases, the stability of the system needs to be strengthened [106]. Therefore, it has been very important to develop a stable structure of a Cu-supported catalyst in this direction. Sarkar et al. [107] synthesized the catalyst Cu@C-POP by FC reaction with catechol and dimethoxymethane. Figure 8a shows the as-synthesized catechol-functionalized C-POP. Copper nanoparticles were then fixed to the polymer by the reduction deposition of two copper salts, respectively. The C-POP had a stable framework and formed a space structure that restricted the agglomeration and leaching of the copper nanoparticles. The system achieved 98% selectivity of DHMF at 150 °C and 20 bar H_2_ for 10 h, and the catalyst could be reused seven times. Figure 8b shows the reaction energy distribution of the hydrogenation process of HMF conversion to DMHF. In addition to the conversion process at the location, we can also see the presence of Cu vacancy on the catalyst surface. The presence of vacancy increased the electron density of the CuO surface, thus improving the adsorption capacity of the substrate molecules.

In particular, 5-ethoxymethylfurfural (EMF), the etherification product of HMF not mentioned in the Figure 1, was identified as an important substance for the preparation of biodiesel and can produce a change from the currently used petroleum-based energy systems [108]. Wang et al. used the soft template method to synthesize ordered mesoporous carbon (OMC) from resorcinol and pluronic F-127, and then sulfonated it to produce a catalyst, OMC-SO_3_H. The fructose was converted into EMF with a yield of 55.7% by a one-pot method [109]. On this basis, Zhang et al., used sulfonic acid functionalized HCPs instead of OMC as the catalyst for the direct reaction of the one-pot method into a mixed solvent system of ethanol and DMSO and obtained EMF and HMF with yields of 78.9% and 15.4%, respectively, by reaction at 104.85 °C for 480 min. The activity of the catalyst HCP-x did not decrease significantly after five catalytic cycles [110]. In this reaction, the high-density sulfonic acid groups and the stability of HCP-based catalysts ensured the activity of fructose etherification. Its abundant Brϕnsted acidic site and high mobility given by its structure guaranteed the efficient conversion of the product with the addition of Lewis acid.

**Figure 9:**
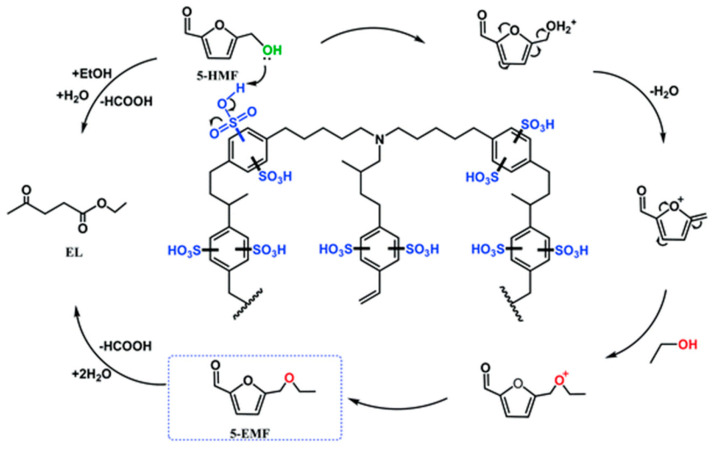
Reaction mechanism of the catalytic conversion of HMF on PDVTA-SO_3_H [112].

Sulfonic acid site-containing POPs catalysts and surface properties that can be regulated as required have great potential for biomass conversion. In the synthesis of EMF, fructose as a raw material did not avoid the problems of time consumption and low efficiency, while using HMF as a raw material shortened the reaction time and achieved higher selectivity and even solvent-free conversion, which is more valuable and feasible from the perspective of green sustainable chemistry [111]. There are various synthetic methods, and most kinds of CMPs combined with sulfonic acid groups also have some application space in the catalytic conversion of HMF. Xiang et al. used divinylbenzene (DVB) and triallylamine (TAA) to synthesize the porous organic polymer PDVTA with a CMP structure by solvothermal copolymerization. The catalyst PDVTA-SO_3_H was obtained after sulfonation. As shown in Figure 9, the structure of the sulfonated CMPs provided a large number of reactive sites, and HMF was etherized into EMF under the action of these acidic sites. The catalytic system, with a 110 °C reaction for 30 min, achieved an 87.6% EMF yield and could maintain five catalytic cycles [112].

**Figure 10:**
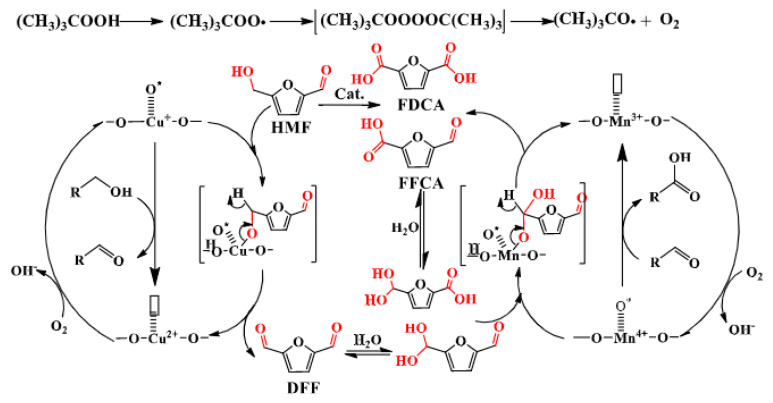
Mechanism for the oxidation of HMF to FDCA catalyzed by Cu-MnO_2_@PDVTA [113].

In addition to the functional modification of POPs, supported catalysts prepared with active components achieved higher performance than the catalysts composed of only active components. In the utilization of PDVTA, Lai et al. loaded Cu-doped MnO_2_ nanowires to prepare Cu-MnO_2_@PDVTA, which achieved up to a 96.8% FDCA yield at 80 °C for 12 h, and the catalyst achieved seven cycles [113]. Figure 10 shows the reaction mechanism of HMF oxidation to FDCA. The conversion of HMF to DFF and FFCA also occurred in the absence of a catalyst, and the FFCA was gradually transformed into FDCA under the action of Mn^4+^. In addition, the doping of Cu^2+^ in the catalyst was conducive to the exchange between Mn^3+^ and Mn^4+^. It also promoted the transfer of lattice oxygen. Since the addition of alkali is required in most non-precious metal catalytic systems, here, they took advantage of the excellent performance of a Mn-based catalyst, considered the influence of its crystal structure on the catalytic oxidation of HMF [114], and improved the stability of the catalyst by using the CMP structure to provide more basic groups so as to realize the alkyl-free catalysis.

Compared to other heterogeneous catalysts, the advantage of CMPs lies in its highly conjugated framework, which effectively reduced the use of metals in conversion, but it was difficult to remove residual trace metal substances in the process of catalyst preparation [115].

In addition to performing functional modifications, POPs can achieve different functions through the composition of monomers. For example, in order to improve the adsorption capacity of CO_2_, Modak et al. used porphyrinic molecules to react with phenyldialdehyde and synthesized the materials with a surface area of 875 m^2^/g through a one-pot method [116]. Saha et al. used this strategy in the preparation of a catalyst for HMF conversion. They used pyrrole and phenylaldehydes after distillation as precursors, and added ferric chloride to acetic acid mediated through hydrothermal synthesis to obtain an Fe^III^-POP-1 catalyst. In this reaction, 100% conversion of HMF and a 79% yield of FDCA could be obtained by using only water as a solvent and reacting in air at 100 °C for 10 h. The catalyst maintained activity in three reaction cycles [117]. No product appeared in the system after the subsequent reaction with a homogeneous Fe^III^–porphyrin complex, which indicates the importance of a large surface area and the multiple active sites produced by this simple and rapid synthesis method for catalytic reaction, and also reflects the interoperability of the two fields of adsorption and catalysis.

Table 2 summarizes the thermal catalytic system of HMF with POPs as the carrier. All the cases listed achieved a high conversion rate of 95% or above under mild reaction conditions. In the case of the same oxidant, different solvents and substrates had large differences in the time required to convert to the target product. However, for the conversion of HMF, whether using oxidation, etherification, or other conversion routes, there have been relatively few studies on POPs as the catalyst. In terms of the current relatively mature thermal catalytic system of HMF, POPs still have a long way to go in terms of their overall catalytic efficiency compared to other porous materials (such as zeolite) with easier access.

## 5. Conclusions and Outlook

It is one of the important tasks of sustainable development to rationally utilize existing energy and develop renewable energy instead of fossil energy. Biomass resources are considered important raw materials to replace fossil-based resources in the production of fuels and chemicals, and can be utilized reasonably on a large scale through catalysts with specific catalytic activities. As described above, POPs have established their position in the field of catalysis by virtue of their permanent pore structure, excellent stability, and low density structure, and they have also demonstrated their great potential in biomass utilization. The functional groups existing on the surface of POPs can provide reaction sites and have the potential to replace metal-based catalysis. At the same time, the stable structure also guarantees the recovery rate of the catalyst and saves costs. Their functionalization is not limited to one or some groups, but with the existing synthesis technology, achieving stable, uniform, and abundant active sites still needs more in-depth research.

This paper mainly reviews the synthesis and the structural characteristics of POPs materials focused on the catalytic application of POPs as the main components of catalysts in lignocellulose conversion to HMF and derivates. In the preparation of HMF by the catalytic dehydration of fructose and the preparation of follow-up chemicals by catalytic HMF, efficient production has not only been achieved through effective functionalization combined with the unique structure of the carrier materials but also through innovative synthesis methods to improve the inherent shortcomings of some POP materials, and their unique design takes into account the convenience of follow-up operations. In addition, the feasibility of the innovative application of some POPs materials is also discussed.

POPs have been widely used in the field of catalysis and have good effects, but their potential has not been fully realized in the catalytic conversion of biomass studied in this paper. To be specific, they basically adopt thermal and electric catalytic systems. Although they have suitable structures for other catalytic systems, representative results in biomass conversion attempts have not yet been seen. We propose to make breakthroughs in the catalytic conversion of biomass using the design of the topological structure, the orderly regulation of the micro-morphology and the pore structure, and the directional loading of functional groups and active sites.

In the practical application of natural resources in synthetic products, the one-step method can avoid the lengthy separation process and the purification process of intermediate compounds in subsequent processing. Cellulose can be converted into alcohol compounds, ester compounds, and organic acid compounds by homogeneous and heterogeneous catalytic systems in the one-pot method. However, POPs as a catalyst component have not been reported. In addition, almost all existing POP materials are synthesized under solvothermal conditions, and their requirements for synthesis environment and equipment make it hard to achieve large-scale synthesis. Although heterogeneous catalysis has improved the separation and purification efficiency of products, the applications of POPs in biomass conversion still face great challenges in separation and purification. We need to accurately extract each of the high-value chemicals from the resulting mixture to produce a product that meets the standards.

Therefore, full use should be made of sustainable energy and technology combined with the structural characteristics of POP materials to continuously innovate and developing an efficient catalytic system so that it cannot only achieve the catalytic conversion into small molecules but also the direct conversion of biomass macromolecules into high-value chemicals. Further, it is of great research significance to meet the needs of the times and develop green and harmless solvent systems and synthesis methods with high industrial compatibility to meet the needs of more fields.

## Figures and Tables

**Table 1:** Summary of reactive systems for the conversion of fructose into HMF using POPs as catalysts.

Catalyst	Substrate	Solvent System	Time (min)	Temperature (°C)	X_substrate_ (%)	HMF Yield (%)	Reference
TFP-DABA	Fructose	DMSO	60	100	>99	97	[76]
PVP@[SO_3_H]_0.17_-COF	Fructose	Tetrahydrofuran	30	100	>99.5	97.6	[77]
POPDS	Fructose	DMSO	20	140	98	86	[78]
POPSDS	Fructose	DMSO	20	140	98	89	[78]
HO_3_S-POP	Fructose	Dioxane aqueous	15	140	100	>70	[81]
STrzDBTH	D-Fructose	DMSO	20	140	100	96.2	[82]
HCP-x	Fructose	DMSO	30	139.85	>99	96.7	[83]
SPPTPA-1	Fructose	DMSO	20	140	100	94.6	[85]
FeSPPTPA	Fructose	DMSO	20	100	95	96.6	[86]
B-POP	Fructose	DMSO/ Dioxane	30	130	100	85	[88]

**Table 2:** Summary of reactive systems for HMF conversion using POPs as catalysts.

Catalyst	Substrate	Solvent System	Oxidant	Pressure (Mpa)	Time (min)	Temperature (°C)	X_substrate_ (%)	Yield (%)	Reference
HCP-x	Fructose	Ethanol/DMSO	Air	0.1	480	104.85	99.8	78.9 (EMF); 15.4 (HMF)	[110]
PDVTA-SO_3_H	HMF	Ethanol	Air	0.1	30	110	99.8	87.5 (EMF)	[112]
Cu-MnO_2_ @PDVTA	HMF	Tertbutyl alcohol	TBHP /Air	0.1	720	80	95	96.8 (FDCA)	[113]
Fe^III^-POP-1	HMF	Water	Air	1	600	100	100	79 (FDCA)	[117]

## Data Availability

Not applicable.
